# Supplementary figures and images for: Alcohol dependence promotes systemic IFN-γ and IL-17 responses in mice
Source: PLoS One. 2020 Dec 21;15(12):e0239246. doi: 10.1371/journal.pone.0239246 (PMC7751976; doi:10.1371/journal.pone.0239246)

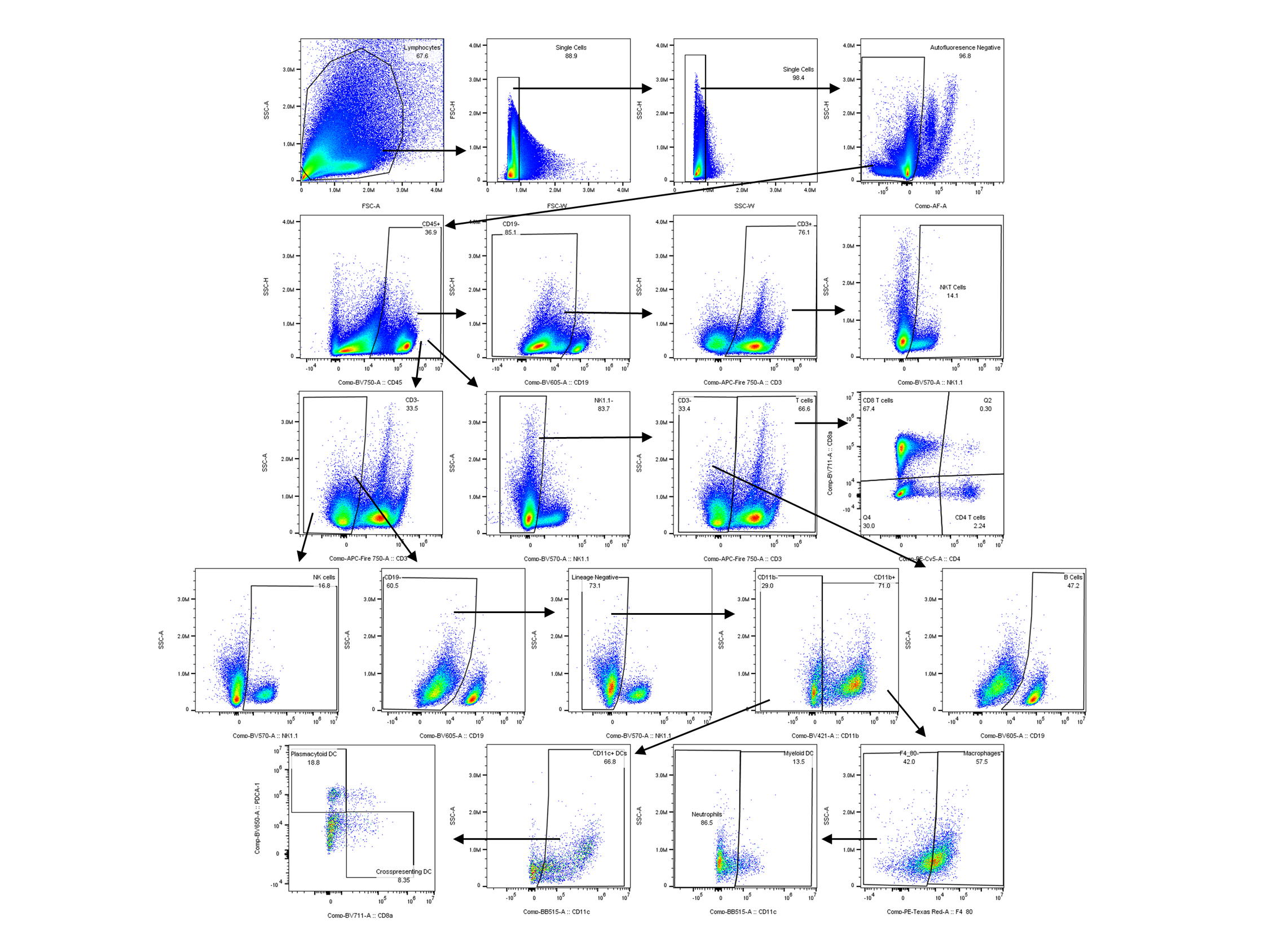

Supplement: S1 Fig — (TIF) [file pone.0239246.s001.tif]

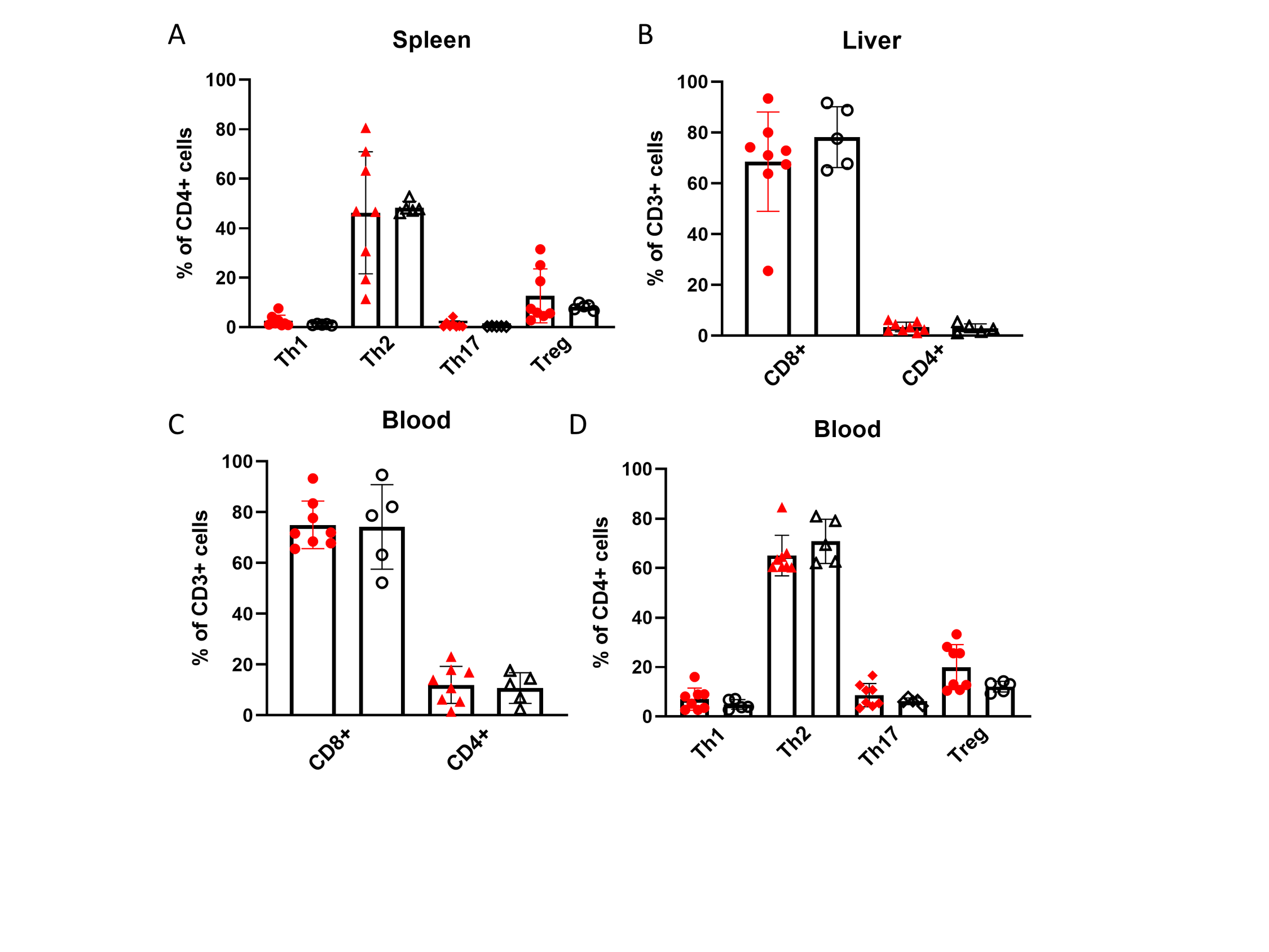

Supplement: S2 Fig — (A) Th1 (CD4+ IFN-γ+), Th2 (CD4+ IL-4+), Th17 (CD4+ IL-17+), and Treg (CD4+ FoxP3+) expression as a percentage of CD4+ T helper cells isolated from spleen of alcohol dependent (red, solid fill) and non-dependent (black outline, white fill) mice. (B-C) CD4+ and CD8+ T cell expression as a percentage of CD3+ cells isolated from (B) liver and (C) blood of dependent and non-dependent mice. (D) Th1, Th2, Th17 and Treg expression as a percentage of CD4+ T helper cells isolated from blood of alcohol dependent and non-dependent mice. *, p<0.05; **, p<0.01 analyzed by Mann-Whitney U test; n = 5–8. (TIF) [file pone.0239246.s002.tif]

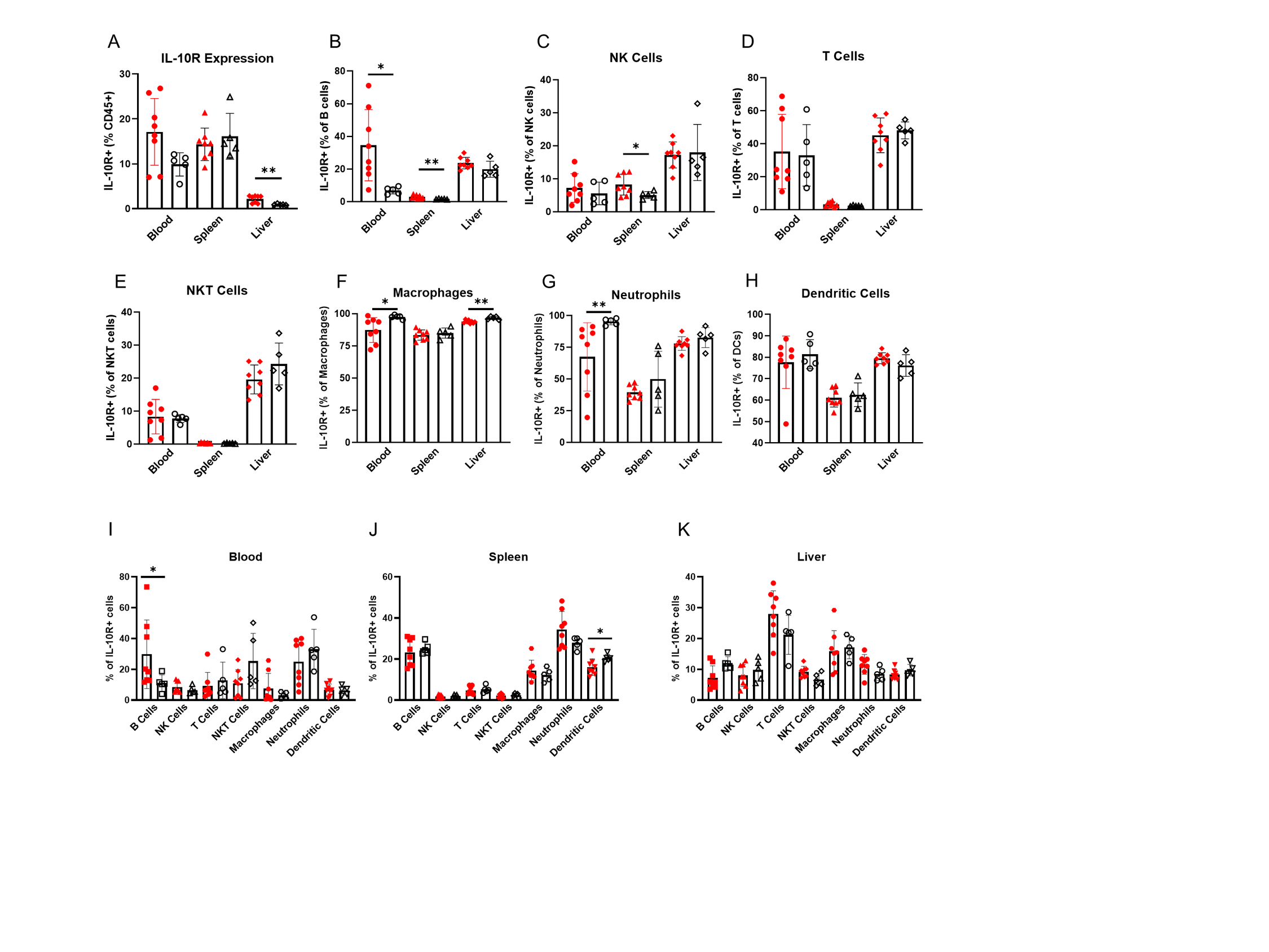

Supplement: S3 Fig — (A) Expression of IL-10 receptor (IL-10R) blood, spleen and liver CD45+ cells isolated from alcohol dependent (red, solid fill) and non-dependent (black outline, white fill) mice. (B-D) Immune cells as percentages of all IL-10R+ CD45+ cells in (B) blood, (C) spleen and (D) liver isolated from dependent and non-dependent mice. (E-K) Expression of IL-10R by (E) B cells, (F) NK cells, (G) T cells, (H) NKT cells, (I) macrophages, (J) neutrophils and (K) dendritic cells in dependent and non-dependent mice. *, p<0.05; **, p<0.01 analyzed by Mann-Whitney U test; n = 5–8. (TIF) [file pone.0239246.s003.tif]

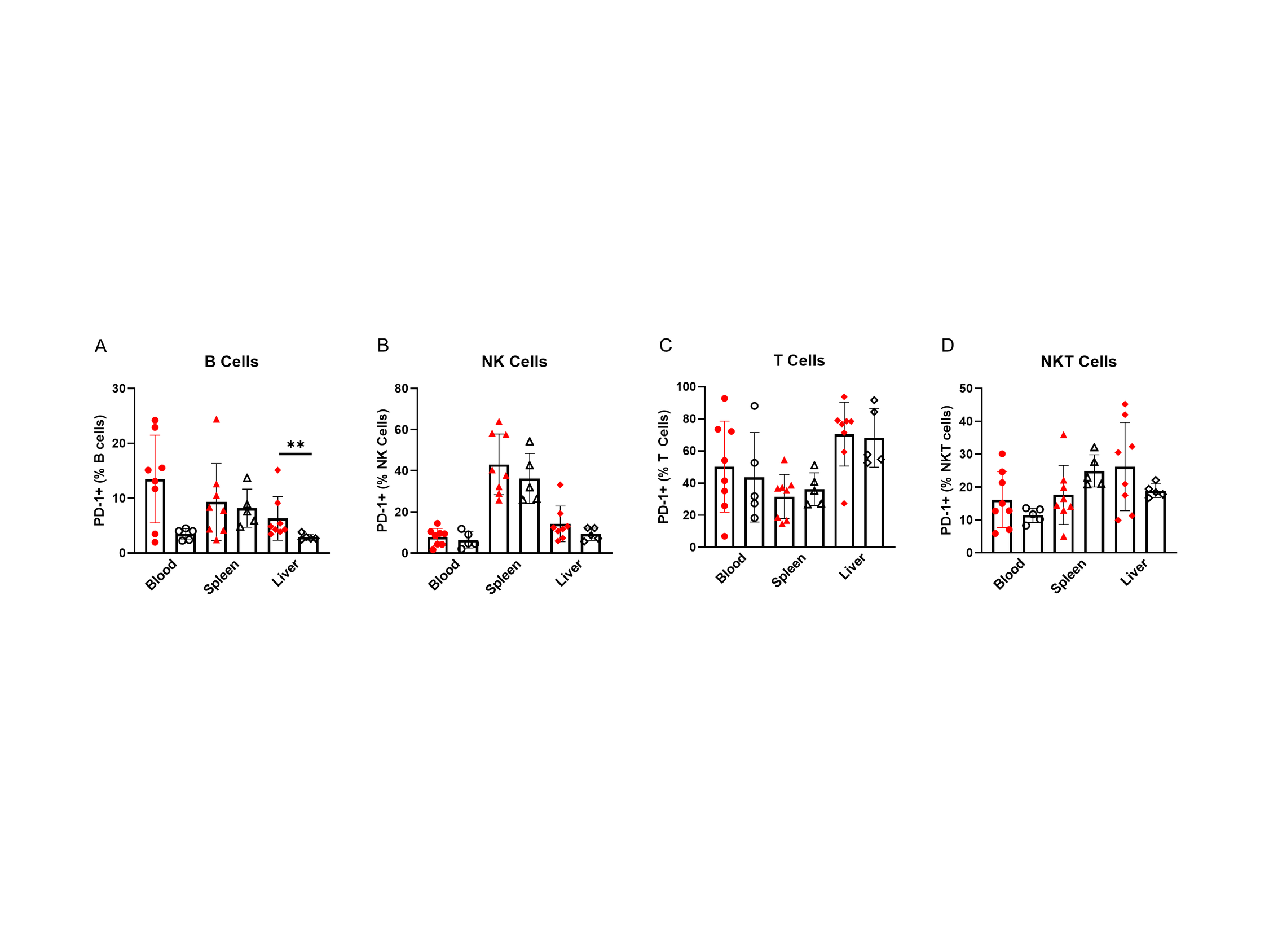

Supplement: S4 Fig — (A-D) Expression of PD-1 by (E) B cells, (F) NK cells, (G) T cells, and (H) NKT cells in alcohol dependent (red, solid fill) and non-dependent (black outline, white fill) mice. *, p<0.05; **, p<0.01 analyzed by Mann-Whitney U test; n = 5–8. (TIF) [file pone.0239246.s004.tif]

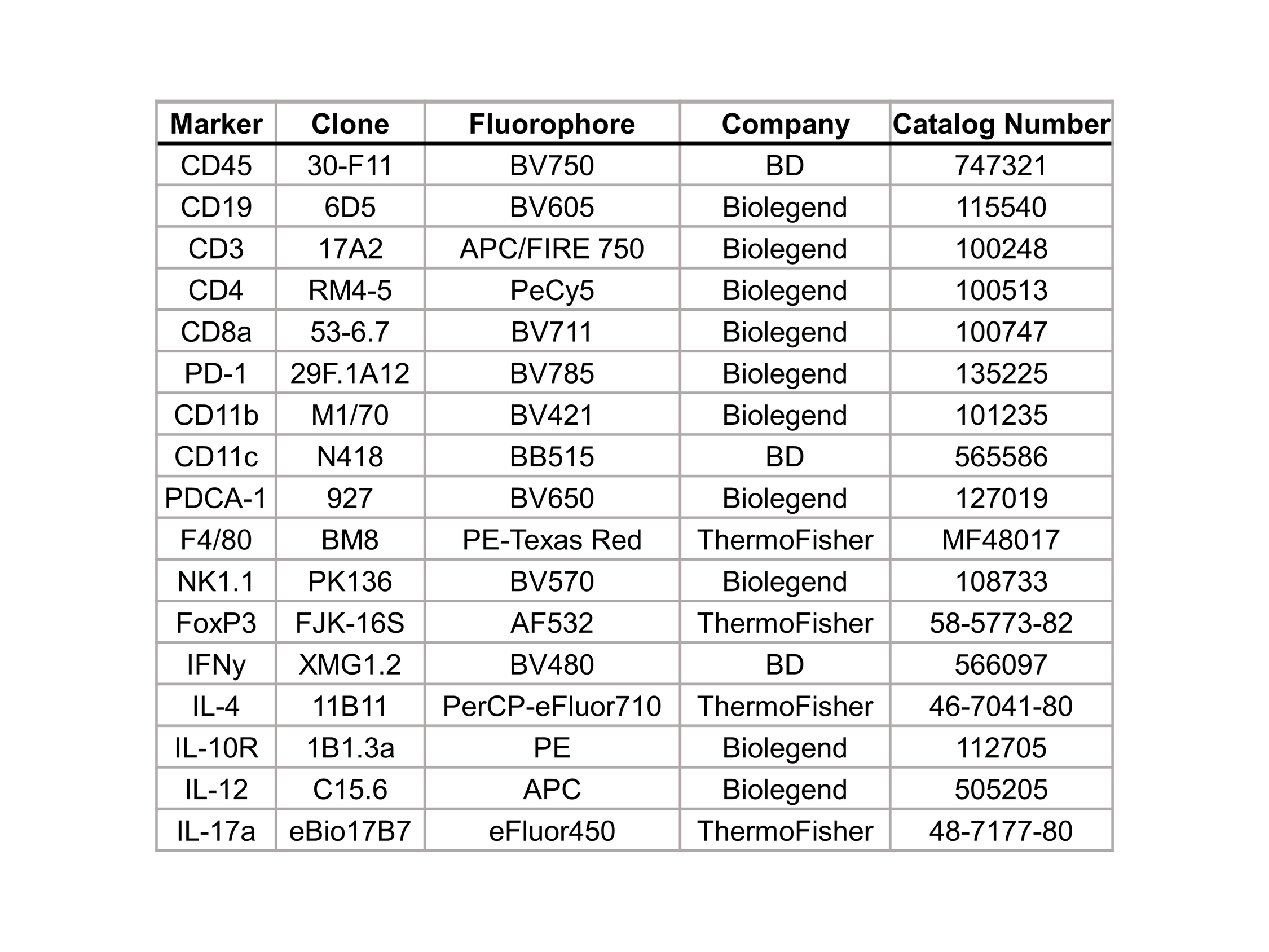

Supplement: S1 Table — (TIF) [file pone.0239246.s005.tif]
